# Inflammatory Bowel Disease and Endometriosis: Diagnosis and Clinical Characteristics

**DOI:** 10.3390/biomedicines12112521

**Published:** 2024-11-04

**Authors:** Mariasofia Fiorillo, Benedetto Neri, Roberto Mancone, Consuelo Russo, Federica Iacobini, Sara Concetta Schiavone, Elena De Cristofaro, Stefano Migliozzi, Caterina Exacoustos, Livia Biancone

**Affiliations:** 1 Gastroenterological Unit, Department of Systems Medicine, University “Tor Vergata” of Rome, 00133 Roma, Italy; fiorillo93@gmail.com (M.F.); benedettoneri@gmail.com (B.N.); roberto.mancone@yahoo.it (R.M.); saraschiavone27@gmail.com (S.C.S.); elena_decr@hotmail.it (E.D.C.); stefano.migliozzi946@gmail.com (S.M.); 2 Therapeutic GI Endoscopy Unit, Fondazione Policlinico Universitario Campus Bio-Medico, 00128 Rome, Italy; 3 Obstetrics and Gynecological Unit, Department of Surgical Sciences, University “Tor Vergata” of Rome, 00133 Rome, Italy; crconsuelorusso@gmail.com (C.R.); fiacobini93@gmail.com (F.I.); caterinaexacoustos@tiscali.it (C.E.); 4 Department of Women, Children, and Public Health Sciences, Fondazione Policlinico Universitario A. Gemelli IRCCS, 00168 Rome, Italy

**Keywords:** inflammatory bowel disease, Crohn’s disease, ulcerative colitis, endometriosis, deep infiltrating endometriosis (DIE)

## Abstract

**Background/Objectives:** Endometriosis and inflammatory bowel disease (IBD) share some epidemiological, clinical and pathogenetic features. A differential diagnosis between pelvic endometriosis and IBD may be challenging, even for expert clinicians. In the present review, we aimed to summarize the currently available data regarding the relationship between endometriosis and IBD and their possible association. **Methods:** The PubMed and Scopus database were considered, by searching the following terms: “Crohn’s Disease”, “Ulcerative Colitis”, “Endometriosis”, “Adenomyosis”, and “Inflammatory Bowel Disease”, individually or combined. Full-text papers published in English with no date restriction were considered. **Results:** Few studies have researched the possible association between endometriosis and IBD. Both conditions are characterized by chronic recurrent symptoms, which may be shared (abdominal pain, fatigue, infertility, menstrual irregularities, diarrhea, constipation). Deep infiltrating endometriosis (DIE) can cause bowel symptoms. In a large Danish study, a 50% increased risk of IBD was observed in women with endometriosis. A missed diagnosis of endometriosis and an increased risk of endometriosis has been reported in IBD. Current evidence does not support an association between endometriosis and IBD characteristics. However, IBD may be associated with DIE, characterized by pelvic symptoms (dyschezia, dyspareunia). Preliminary observations suggest an increased IBD risk in patients with endometriosis treated with hormonal therapy. **Conclusions:** Current findings suggest that a careful search is needed for concomitant endometriosis in subgroups of patients with IBD showing compatible symptoms and vice versa. A multidisciplinary approach including dedicated gastroenterologists and gynecologists is required for a proper search for IBD and endometriosis in subgroups of patients. This approach may avoid diagnostic delays or overtreatments for these conditions.

## 1. Introduction

Endometriosis and inflammatory bowel disease (IBD) share some epidemiological, clinical and pathogenetic features. Supporting this concept, both diseases typically affect the young population and show a chronic relapsing course.

Recently, both conditions showed an increasing incidence in childbearing age. Overall, endometriosis is quite frequent, being observed in up to 10% of premenopausal women [1].

Endometriosis and IBD may involve the same organs (intestinal localization of endometriosis observed in 5–12% of women), thus determining the occurrence of similar symptoms (abdominal and/or chronic pelvic pain, change in bowel habits). Moreover, even though uncommon, ileal and sigmoid endometriosis may mimic Crohn’s disease (CD) with stricturing behavior, as it may present with subocclusive symptoms. More importantly, chronic recurrent abdominal pain is a characteristic symptom occurring in both patients with IBD (particularly CD) and in patients with endometriosis. In both conditions, disease onset is typically observed at a young age [2]. Due to these overlapping symptoms, a differential diagnosis between pelvic endometriosis and IBD may be particularly challenging, even for expert clinicians. Based on these observations, the relationship between these two conditions has been investigated during the last few decades, although studies in this respect are currently limited.

Concomitant endometriosis and IBD has been initially described since the early 2000s [3]. In both diseases, a dysregulation of the immune system has been described in the affected patients. In terms of pathogenesis, several immunological features similar to those observed in IBD and other autoimmune diseases have been reported in patients with endometriosis [4,5].

A mildly increased incidence of autoimmune diseases, such as multiple sclerosis, systemic lupus erythematosus, Sjogren’s syndrome and even celiac disease have been described in endometriosis patients [6]. Differently, the association with IBD has not yet been convincingly demonstrated. This issue assumes relevance mainly in relation to the possible underestimation of concomitant endometriosis in patients with a previous diagnosis of IBD, thus leading to inappropriate treatments. Less frequently, IBD is erroneously diagnosticated in patients with endometriosis involving the intestine. Whether treatments for IBD or for endometriosis in patients with both conditions may influence the course of these two diseases is also currently undefined.

In order to investigate this issue, the aim of the present review is to summarize the currently available data regarding the relationship between endometriosis and IBD and their possible association.

## 2. Materials

For the aim of this narrative review, the PubMed and Scopus database were considered, by searching the following terms: “Crohn’s Disease”, “Ulcerative Colitis”, “Endometriosis”, “Adenomyosis”, and “Inflammatory Bowel Disease”, either individually or combined. The search was focused on full-text papers published in English with no date restriction. Original articles, case-series and case-reports including data from patients with concomitant endometriosis and IBD were reported. For the aim of this narrative review regarding case-series and case-reports, only studies including >7 patients with IBD with concomitant endometriosis diagnosed according to the below reported standard criteria were considered. The studies involving patients with IBD and/or endometriosis are summarized in Table 1.

## 3. Relevant Sections

### 3.1. Inflammatory Bowel Disease

IBD includes ulcerative colitis (UC) and CD. In up to 10% of cases, CD cannot be distinguished from UC, and IBD is therefore classified as IBD-unclassified [2,15,16]. IBD are multifactorial complex diseases of unknown etiology. In terms of pathogenesis, a dysregulation of the host immune response toward luminal antigens in genetically susceptible individuals currently appear to play a key role [17]. The epithelial barrier function, the innate and adaptive immunity and the commensal bacteria, with the concurrence of environmental factors, are involved in determining an inappropriate immunoinflammatory response [17]. The activation of the immune system in the damaged tissue may cause an inappropriate release of a cascade of anti- and proinflammatory signals [18]. CD and UC share several inflammatory pathways, as supported by the well-defined responsiveness to the same immunomodulatory drugs specifically targeting mediators involved in the pathogenesis of tissue damage (i.e., TNFα and IL12/IL23 antagonists). Nevertheless, macrophages and T cells activation leading to higher levels of IL-1, IL-12, interferon-γ and IL-18 is more typically observed in CD gut [19,20]. Among factors contributing to the development of tissue damage in IBD, attention has recently been focused on a dysregulation of the mucosal immune response toward the gut microbiota and its interaction with the epithelial barrier [21]. Differences in the gut microbiome have indeed been described in patients with IBD when compared to the general non-IBD population. A higher degree of dysbiosis has been reported in patients with CD than with UC [22,23]. Supporting this concept, lower levels of *Eubacterium rectale*, *Faecalibacterium prausnitzii* and *Roseburia intestinalis* in IBD and of *Bifidobacterium longum* (only in UC) have been reported. Differently, the relative abundance and growth rate of harmful bacteria such as Bacteroides fragilis have been described in IBD [24].

Recently, the involvement of lipids in the pathogenesis of IBD has been suggested. Indeed, lipids are responsible for cell membrane integrity and intercellular signaling. The disturbance of these functions significantly affects the expression and maintenance of inflammation and thus may contribute to the development of tissue damage in IBD [25]. These diseases are also known to be associated with other dysimmune diseases such as spondyloarthritis, erythema nodosum, pyoderma gangrenosum, uveitis and psoriasis [26,27,28].

On the basis of the reported evidence, therapies targeting inflammatory pathways have been developed, including monoclonal antibodies and, more recently, small molecules. The first monoclonal antibody developed for treatment of IBD was the anti-TNFα infliximab (first trial 1995) followed by adalimumab [29]. TNFα released by activated T lymphocytes and macrophages may indeed stimulate the acute phase response, promote the secretion of cytokines mostly provided of proinflammatory activity (i.e., IL-1, IL-6, IFN-γ) and increase the expression of adhesion molecules. Overall, TNFα is a key mediator of the inflammatory response involved in the pathogenesis of IBD [30]. After infliximab and adalimumab, other monoclonal antibodies have been developed for treating patients with IBD, specifically targeting different pathways, including IL-12/IL-23 and the immune cell trafficking in the gut. IL-12 and IL-23 share the subunit p40, are produced by the dendritic cells and are known to be involved in the IBD-related chronic inflammation. These cytokines drive the differentiation, proliferation and activation of immune cells including CD4+ T cells, NK cells, NKT cells and Th17 cells [31]. In the last decade, ustekinumab (anti-IL12/IL-23 monoclonal antibody) has been approved for treating active UC and CD patients [29]. Very shortly, the selective anti-IL23 antibodies mirikizumab and risankizumab will also be available in the market [29]. Differently, vedolizumab (approved for IBD) and natalizumab (approved for CD in the United States), exert their action by targeting adhesion molecules (α4β7 integrin or the α4 subunit, respectively), thus limiting the immune cell migration into the gut [29]. More recently, new oral small molecules have been approved in UC and CD, exerting their activity by targeting the JAK-STAT pathway, among the initial steps of the inflammatory cascade involved in the pathogenesis of IBD [29].

UC is a chronic inflammatory condition affecting the rectum with possible involvement of the entire colon, with typical continuous lesions [16,26,32]. Clinically, UC is characterized by a chronic relapsing course [16,26,32]. UC onset typically occurs at a young age, during the second or third decades of life [5,24,32]. Diagnosis of UC is based on a combination of gastrointestinal symptoms, biochemical markers and endoscopic and histological findings [15,16,32]. Symptoms related to UC always include bloody diarrhea and rectal bleeding, frequently associated with tenesmus, urgency and fecal incontinence. Abdominal pain, including pelvic pain may occur in patients with moderate-to-severe UC. Bowel movements occurring at night, fatigue, iron-deficiency anemia (IDA) and weight loss may be observed. Abdominal pain, anorexia, fever, severe IDA and hypoalbuminemia all suggest severe colitis [16,32,33]. UC severity is related to the extent of the lesions. Complications, including toxic megacolon, perforation, massive bleeding from the colon and multiple organ dysfunction (MOF) may develop in a subgroup of UC patients (<10%), particularly in the case of severe extensive colitis [16]. One main issue in patients with UC includes the risk of developing colorectal cancer (CRC) [34]. CRC risk is significantly increased in UC patients with a long-standing (≥8 years duration), extensive UC characterized by a chronically active course, severe colonic lesions, history of adenomatous polyps or familial CRC [35,36]. A concomitant diagnosis of primary sclerosing cholangitis represents the main risk factor for CRC in UC, and therefore a surveillance colonoscopy every year is indicated in patients with both conditions [16].

Typical endoscopic findings of UC include erythema, loss of normal vascular pattern, granularity, erosions, friability, spontaneous bleeding and ulcerations [37]. No specific histological features per se allow a certain diagnosis of UC. Typical histological alterations in UC include crypt distortion, branching and shortening, increased lymphocytes and plasma cells in the lamina propria (basal plasmacytosis), mucin depletion, crypt abscesses and Paneth cell metaplasia [38,39]. Therefore, for a proper diagnosis of UC, ileocolonoscopy with biopsies is mandatory, unless in patients with severe ulcerative colitis (ASUC), due to the risk of iatrogenic colonic perforation. In these patients, sigmoidoscopy may be performed by experienced endoscopists [16].

First-line therapy in mild-to-moderate UC include aminosalycilates treatments, given by mouth or by enema [40,41,42]. Oral corticosteroids represent the first treatment strategy for patients with mild-to-moderate UC despite aminosalycilates. Corticosteroids are not indicated for maintaining remission, due to the well-known risk of side effects [40,41,42]. In moderate-to-severe UC, thiopurines (azathioprine or 6-mercaptopurine), biologics (anti-TNFα, anti-IL-12/23, anti-α4β7 integrins) and small molecules (JAK inhibitors) represent highly effective strategies [40,41,42]. Treatments need to be chosen on a patients’ basis.

CD is a chronic disease, potentially affecting the entire gastrointestinal tract, although the terminal ileum and colon represent the more frequently involved areas. Inflammation is typically segmental, asymmetrical and transmural. CD occurs at a young age, as described for UC [43,44]. Up to one-third of patients with more severe CD show strictures, fistula or abscesses at diagnosis. However, most patients develop complications during the disease course, with roughly 50% of them requiring surgical treatment within the first 10 years after diagnosis [45]. After “curative” intestinal resection, postoperative recurrence of the lesions is a typical characteristic of CD [45].

CD diagnosis relies on a combination of clinical history, signs, symptoms and radiologic, endoscopic and/or histological findings [2,38,45]. Symptoms related to CD show wide interindividual variations, being related to the site, severity and type of the lesions [45]. The most common scenario at CD onset includes a young patient with a history of chronic recurrent abdominal pain, associated with chronic diarrhea and weight loss. Fatigue and anorexia are common symptoms too and fragility may occur [45,46]. Signs and symptoms related to malabsorption typically occur in CD patients with small bowel involvement of the lesions. In patients with colonic involvement, rectal bleeding or bloody diarrhea may occur. Low-grade fever is a common symptom, while high-grade fever may indicate the development of abdominal abscesses. Approximately one-third of patients develop perianal disease, occurring at CD onset in subgroups of patients [45,47]. Complications in CD include obstruction, abdominal abscess, perforation and gastrointestinal hemorrhage [48]. CRC risk is increased in CD patients with colonic involvement, particularly in patients with a long history of colonic strictures [34]. The risk of developing small bowel adenocarcinoma, although rare, is markedly increased in CD patients with small bowel involvement of the lesions, particularly in the presence of long-standing strictures at this level. The diagnosis and surveillance of SBA in CD may be clinically challenging in relation to difficulties related to biopsy sampling, particularly in small bowel areas not reachable by the endoscopes.

Typical endoscopic findings in CD include segmental inflammation and ulcers (aphthoid, longitudinal, serpiginous) [37,45]. A typical cobblestone pattern is frequently observed, being related to deep ulcerations, surrounded by nodular edematous mucosa. Stenosis, fistulae and abscesses may also be observed in complicated CD.

In CD, histological findings typically include a chronic focal, patchy, discontinuous and transmural inflammatory infiltrate with goblet cell preservation. T cell and macrophage infiltration, mainly in the submucosal layer (“disproportion”) is a feature of the involved CD lesions [39]. Transmural lymphoid aggregates and pyloric gland metaplasia are also common findings. Only one histological hallmark may be rarely observed in the submucosal lesions of patients with CD. This is the epithelioid granuloma, detected in only about 15% of mucosal biopsies and in up to 70% of surgical specimens, due to its deep location in the affected bowel wall [38,39,45]. Therefore, there is no need to search for granuloma in order to make a diagnosis of CD.

Overall, as for UC, ileocolonoscopy with biopsies of inflamed and uninflamed segments is mandatory for a well-defined diagnosis of CD. However, different to UC, in patients with suspected CD, cross-sectional imaging, currently represented by small bowel ultrasonography and entero-CT or entero-RM, plays a pivotal role for both diagnosis and staging. Indeed, even in cases of absence of pathological findings at ileocolonoscopy, the small bowel should be evaluated with cross-sectional imaging [15].

Several treatment strategies are available for treating CD. Aminosalycilates may be used for preventing CD recurrence or for maintaining clinical remission, although data in this respect are conflicting [49,50]. Corticosteroids (systemic or low absorbable) represent the first-line treatment during the active phase of CD. Thiopurines, biologic and small molecules are effective treatments for steroid-refractory or steroid-dependent patients and in moderately-to-severely active CD [49,50]. Treatment choice is related to characteristics of the host, including comorbidities, and of the disease. In both CD and UC, medical treatment should be tailored on a patients’ basis.

Despite the growing number of highly effective treatments for UC and CD, subgroups of patients still represent a major clinical issue even for IBD-dedicated gastroenterologists. Among these, patients with acute severe ulcerative colitis (ASUC) may represent a serious clinical condition difficult to manage. ASUC is indeed the most severe complication of UC, possibly leading to colectomy and even to death. Currently, ASUC treatment relies on hospitalization, high dose i.v. corticosteroids (0.8/1 mg/kg up to 60 mg/daily) and, in case of failure, infliximab [16]. However up to 30% of patients end up with a colectomy within 1 year from the ASUC episode [16]. New small molecules are being tested in this context; however, further data are needed before using alternative treatments for this feared UC complication. In clinical management of CD, one of the main issues is represented by the high rates of treatment failure, ranging up to 30–40% of patients during the first year of anti-TNFα treatments [51]. Primary or secondary failure to current medical treatments accounts for most of the indications for CD-related surgery, required in up to two-thirds of these patients [52]. In CD, the postoperative recurrence, including the new appearance of the intestinal lesions after surgery, also represent a major issue. No treatments are indeed currently available in order to prevent the recurrence of CD lesions, thus leading to chronicity [50].

Thus, for both UC and CD, it is imperative to further investigate therapeutic options and treatment algorithms in order to improve the clinical outcome and the quality of life of the affected patients.

### 3.2. Endometriosis

Endometriosis is an inflammatory, estrogen-dependent condition defined by the presence of endometrial glands and stroma in ectopic locations. It is common among women, affecting 6–10% of those in childbearing age. In the subpopulation of symptomatic and infertile women, its prevalence is particularly high, ranging from 35% to 50% [53].

The disease is generally more common in highly developed countries, among higher socioeconomic groups and within the European population [54,55]. Indeed, the incidence and prevalence of endometriosis has been reported to show significant variations across different countries, likely due to disparities in healthcare access, diagnostic capabilities and genetic or environmental factors. A recent retrospective cohort study using electronic health records estimates that 70% of patients with a diagnosis of endometriosis are Caucasian, 6% Hispanic, 9% Asian and 4.7% non-Hispanic Black [56]. In a meta-analysis by Bougie et al., Black and Hispanic women were less likely to be diagnosed with endometriosis than Caucasian women, while Asian women were at a higher risk of this disease [57]. However, it is imperative to recognize the significant methodological flaws and bias driving the studies performed to date. In particular, the perception of a lower risk among Black women was widely spread in past medical literature, although recent data suggest a relevant role played by significant disparities in access to care and diagnosis among different ethnic groups [57]. A higher rate of undiagnosed endometriosis in populations resident in less developed countries may be involved in this finding.

Despite its high occurrence, the prevalence of endometriosis is widely believed to be underestimated. Nonspecific symptoms and the diagnostic challenges in detecting endometriosis are among the main factors contributing to this clinical challenge. Supporting this concept, the diagnostic delay ranges from 5 to 8 years in adult women [58,59], being even higher (≥10 years) at a younger age [60].

Endometriosis is a disease with a still poorly understood etiology, believed to be multifactorial, resulting from the interplay of several factors. These factors include genetic predisposition, immune system abnormalities, environmental influences and anatomical considerations.

Studies have indicated that women who experience early menarche, frequent menstrual cycles (polymenorrhea) and heavy menstrual flow are more likely to develop endometriosis [61]. Recent research also suggests that body mass index (BMI) is associated with the disease, showing that women with endometriosis typically have a lower BMI compared to healthy individuals, and those with a very low BMI are at a higher risk for deep pelvic endometriosis. Additionally, a family history of the condition is another factor linked to both its severity and likelihood of occurrence [62]. Multiple studies have confirmed that certain environmental substances play a role in the development of endometriosis. Notably, 2,3,7,8-tetrachlorodibenzo-p-dioxin and other dioxin-like compounds are implicated. These “endocrine disruptors” contribute to the disease’s development by increasing interleukin levels, activating cytochrome P-450 and causing changes in tissue remodeling [63,64].

Over the years, numerous theories have emerged to explain the disease’s origin, as no single hypothesis has been able to encompass all the aspects of its pathogenesis. However, the metastatic model, which suggests that endometrial cells are implanted in the pelvis through retrograde flow via the fallopian tubes during menstruation, is backed by the most substantial amount of evidence [65]. The core idea is based on the concept of repetitive ovulatory menstruation (ROM), which leads to recurring bleeding and, as a result, acts as a direct or indirect source of iron-related oxidative stress and subsequent chronic inflammation [66]. In physiological conditions, refluxed endometrial tissue is cleared from the peritoneum by the immune system. The exposure to repeated menstruations-related tissue injury and repair and monthly transtubal menstrual reflux may be greater than the female immune system has genetically evolved to handle within the pelvic environment [63,67,68]. An inappropriate NK-cell and macrophage function in women with endometriosis may further contribute to a decreased clearance of the lesions.

Increasing evidence supports to consider endometriosis as a pelvic inflammatory condition. Survival and implantation of the ectopic endometrial tissue has been suggested to be favored by a dysregulation of the host immune response. In healthy subjects, immunocompetent cells such as macrophages and natural killer (NK) cells are responsible for clearing menstrual debris from the pelvic cavity. In women with endometriosis, this activity appears to be impaired. Symons et al. suggested that a reduced function of these cells in patients with endometriosis, may contribute to a proinflammatory environment supporting the development and persistence of endometriotic lesions [69]. Furthermore, a reduced NK cell cytotoxicity has been reported in these patients, leading to a defective clearing of ectopic endometrial cells [69].

Interleukins play a relevant role in the inflammatory environment that characterizes endometriosis, being potentially associated with some of the symptoms, including infertility and pelvic pain. However, while an association between higher IL-6 and/or IL-8 levels and endometriosis-associated infertility has been reported, the same association was not observed for endometriosis-associated pain [70]. These findings emphasize that whether and how these mediators contribute to the pathophysiology of endometriosis-associated infertility or pelvic pain is undefined. Overall, these findings highlight the conceivable complex role of the immune system in endometriosis and suggest that, in the future, targeting immune pathways could be a promising strategy for therapeutic interventions.

Higher levels of cytokines or chemokines have also been described in patients with endometriosis, including macrophage migration inhibitory factor, TNF-α, IL-1β, IL-6 and IL-8 [71,72,73]. Indeed, an increased number of immunocompetent cells and higher levels of cytokines have been reported in the peritoneal fluid of patients with endometriosis [74,75]. Inflammatory cells, the ectopic endometrial implants and peritoneal mesothelial cells synthesize and secrete cytokines, showing relation with endometriosis for most of them. IL-1, stimulating the synthesis of prostaglandins, is increased in the peritoneal fluid of endometriosis patients [76]. IL-6 is a pleiotropic cytokine also secreted by macrophages that promotes endometrial cell proliferation and angiogenesis in patients with endometriosis [69]. IL-8 is a potent angiogenic, proinflammatory and cellular proliferation cytokine, which was also found in the peritoneal fluid, facilitating endometrial cell adhesion [77]. IL-10 may contribute to the dysregulation of the immune response observed in patients with endometriosis, by modulating local inflammation and contributing to remove apoptotic cells then reducing the local inflammatory response [78]. Higher circulating levels of IL-10 have indeed been reported in patients with stage III and IV endometriosis [70].

Moreover, in patients with endometriosis, as also with IBD, a possible role for an alteration of the lipids metabolism has been suggested [79]. Arachidonic acid is a precursor of several proinflammatory mediators including prostaglandins and leukotrienes, modulating the immunoinflammatory response. In patients with endometriosis, a dysregulation of the arachidonic acid metabolism has been suggested. This event has been hypothesized to contribute to the chronic inflammatory process, supporting the survival and growth of ectopic endometrial tissue. Recent findings suggest that these derivatives may not only promote inflammation, but also affect the reproductive processes, mediated by their action on vascular permeability and immune cell recruitment [79]. This highlights the potential of targeting arachidonic acid pathways as a therapeutic approach in managing endometriosis-related inflammation and symptoms. Current evidence reports that in patients with endometriosis, a decreased eicosapentaenoic acid (EPA) to arachidonic acid ratio is correlated with disease severity [80,81]. Overall, low EPA levels and high phospholipase activity have been involved in the inflammatory pathways of endometriosis. Recent research highlighted the potential involvement of the microbiome in the pathogenesis of endometriosis. Salliss et al. discussed the role of both the gut and genital microbiota, as well as the estrobolome, in the development and progression of endometriosis, infertility and chronic pelvic pain [82]. The study suggests that microbiomes currently appear to play a role in the gut–brain axis, which further supports a putative association with the spectrum of symptoms associated with endometriosis, including infertility and chronic pelvic pain. Additionally, the estrobolome, a collection of genes encoding estrogen-metabolizing enzymes in the gut microbiome, may be involved in modulating estrogen levels, thus affecting the hormonal environment that fuels endometriotic lesions.

Although the natural history of endometriosis remains unknown, emerging evidence suggests that its pathophysiological steps of initiation and development must occur earlier in life [83]. Indeed, the onset of endometriosis-associated pain symptoms is most often reported during adolescence and young adulthood [84,85].

Endometriosis may occur in three different forms:

- Ovarian endometriosis, with the typical appearance of an ovarian cyst containing coagulated, chocolate-colored blood (endometrioma).
- Superficial peritoneal endometriosis, with typical superficial lesions (red or brown), with or without adhesions between various pelvic organs.
- Deep infiltrating endometriosis (DIE), defined as a lesion infiltrating the peritoneum and involving the retroperitoneal space or the walls of pelvic organs, larger than 5 mm.

When endometriosis affects the myometrial wall, it is known as adenomyosis or internal endometriosis. This condition, considered a benign invasion, causes the uterus to enlarge diffusely or in a nodular shape. Some researchers include adenomyosis in the mechanisms leading to infertility [86]. Microscopically, adenomyosis is characterized by ectopic endometrial glands and stroma within hypertrophic and hyperplastic myometrial tissue, either diffusely spread or localized in circumscribed, noncapsular cellular aggregates [87].

DIE affects between 4% and 37% of patients with endometriosis. The most affected retroperitoneal area is the retrocervical zone with invasion of torus and uterus sacral ligament (USL). From this retroperitoneal area, DIE can invade the vagina, the rectovaginal septum and the bowel. The presence of ovarian endometriomas is often associated with DIE while adenomyosis is present in up to 50% of patients with this localization of endometriosis.

Bowel endometriosis typically presents as a single nodule, with a diameter larger than 1 cm, commonly infiltrating the muscularis of the bowel and the surrounding structures, with only 10% of patients showing mucosal involvement [88]. Bowel involvement is observed in 5% to 12% of the women with endometriosis, the rectum and sigmoid colon being involved in up to 90% of all intestinal lesions [89,90].

The most frequent signs and symptoms related to endometriosis include dysmenorrhea, dyspareunia, chronic pelvic pain, dyschezia, dysuria and infertility [91]. Moreover, women presenting with pelvic endometriosis frequently show more severe gastrointestinal symptoms during menstruation [92]. Bowel DIE, as also adenomyosis and other DIE lesions located in the retrocervical area, can cause bowel symptoms, since the inflammation at this level affects the near intestinal wall. Adenomyosis often causes dysmenorrhea and heavy menstrual bleeding (HMB).

Endometriosis diagnosis is often challenging, having historically relied on laparoscopy and histological confirmation. More recently, growing evidence supports the relevant role of noninvasive diagnostic tools such as transvaginal ultrasonography (TVS) and pelvic magnetic resonance (MRI) performed by dedicated physicians in diagnosing endometriosis [93,94]. In 2022, the European Society of Human Reproduction and Embryology (ESHRE) guidelines have recognized TVS as a valid diagnostic tool for detecting endometriosis without the need of laparoscopic or histological confirmation [95].

In the advent of TVS- or MRI-enabled noninvasive diagnosis, the noninvasive imaging facilitates the diagnosis of the disease at earlier stages, at a younger age and with lower costs, using a technology with easier access for most patients. The use of diagnostic imaging such as ultrasound (US) or MRI is crucial for an accurate diagnosis. The typical appearance of endometriotic pelvic lesions using TVS, such as endometriomas, DIE and adenomyosis, can indeed be medically treated without histological confirmation. However, in case of an early stage of the disease with superficial invasion and small endometriotic foci, instrumental diagnostic imaging may often not contribute meaningfully to the diagnosis, but symptoms can guide the medical treatment as well.

**Figure 1:**
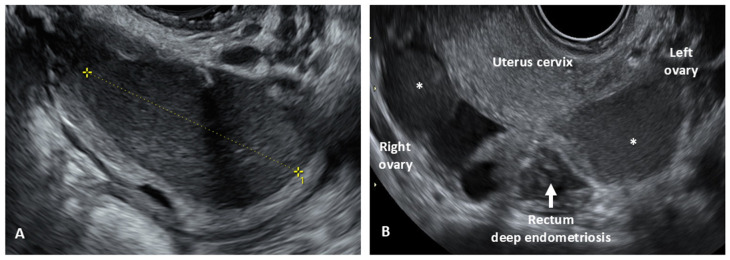
Transvaginal ultrasound (TVS) appearance of ovarian endometrioma. (**A**) A unilocular cystic lesion with smooth walls and a “ground glass” appearance is the most typical presentation of an endometrioma; (**B**) bilateral endometriomas (with asterisks) giving the appearance of “kissing ovaries”.

**Figure 2:**
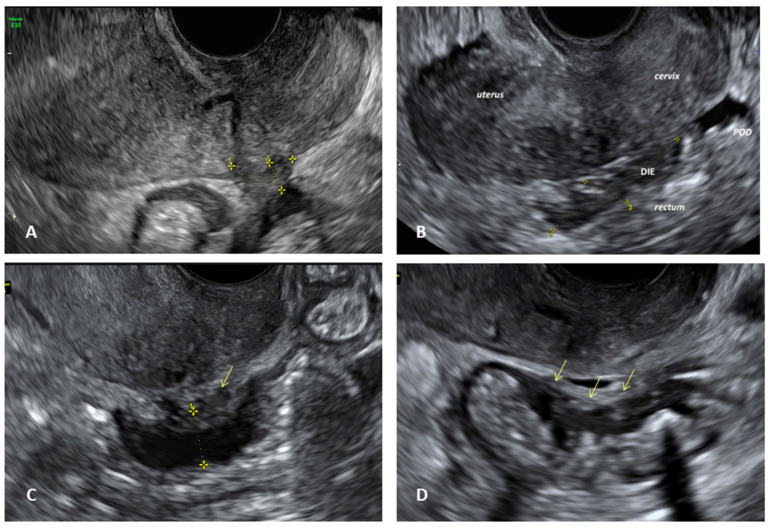
Transvaginal ultrasound (TVS) appearance of deep infiltrating endometriosis (DIE). (**A**) Utero-sacral ligaments (USL) hypoechoic nodule (dotted yellow lines); (**B**) DIE hypoechoic nodule involving rectum and uterine torus (POD: pouch of Douglas); (**C**) rectal endometriosis nodule (dotted yellow lines) and associated USL nodule (yellow arrow); (**D**) rectal endometriosis nodule (yellow arrows).

Recently, a consensus was published on the terms, measurements and definitions to be used for describing the different forms of endometriosis during ultrasound examinations [95] (Figure 1 and Figure 2).

**Figure 3:**
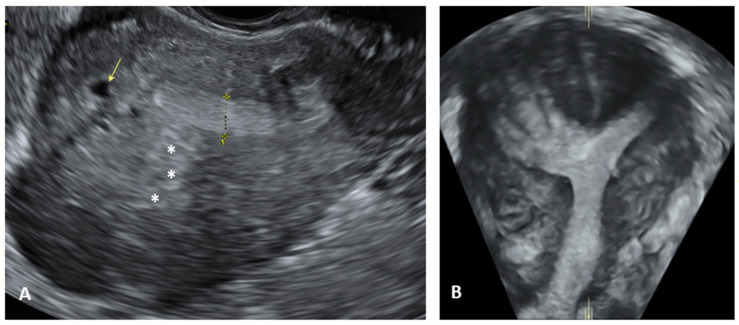
Transvaginal ultrasound (TVS) appearance of adenomyosis. (**A**) Two-dimensional (2D) TVS evaluation shows direct diagnostic signs: myometrial cystic areas (yellow arrow), hyperechoic foci (with asterisks); (**B**) Three-dimensional (3D) TVS evaluation of adenomyosis shows a diffuse undefined junctional zone.

In 2015, a consensus established the ultrasound signs of adenomyosis [96]. In 2022, the MUSA group conducted a review of the previous 2015 consensus and indicated, for the diagnosis of adenomyosis, direct and indirect signs. According to this new classification, the direct signs that diagnose adenomyosis with a high probability are intramyometrial cysts, hyperechoic islands, subendometrial or JZ hyperechoic lines or buds [97] (Figure 3).

Only in patients with negative imaging results or where empirical treatment was unsuccessful or inappropriate, it has been recommended to offer laparoscopy for the diagnosis and treatment of suspected endometriosis. However, a negative histology result does not completely exclude the presence of the disease [83].

Some studies have evaluated the diagnostic accuracy of barium enema and colonoscopy for diagnosing endometriosis and found that neither technique surpasses ultrasound in diagnostic accuracy. Barium enema does not allow for a full investigation of the intestinal wall, and colonoscopy only examines the intestinal mucosa, which is rarely invaded by endometriotic lesions [98]. Therefore, colonoscopy is mainly used for differential diagnosis with other malignant GI tract pathologies [99]. Lastly, in cases of lateral compartment endometriosis involving the ureters and consequently the kidneys, CT urography with contrast medium can be a valuable diagnostic tool, especially in the preoperative phase. Additionally, TVS is essential for the follow-up of patients undergoing medical and/or surgical therapies.

The management of endometriosis should be undertaken in specialized reference centers to provide patients with the necessary expertise and access to multidisciplinary teams essential for this type of pathology. The management of the disease must be tailored considering the symptomatic profiles, the stage of the disease and the patient’s expectations, taking into account surgical risks, limitations of minor procedures and long-term benefits. The choice of treatment is closely linked to the severity and symptoms reported by the woman, the location of endometriotic lesions and the woman’s desire to preserve fertility. The two main therapeutic goals are the reduction or elimination of pain symptoms and the maintenance of fertility.

In recent years, therapeutic strategies for treating endometriosis have shifted from surgery as the first choice to medical treatments aimed to induce amenorrhea. This order is to allow a maximal preservation of the ovarian reserve in childbearing women. Therefore, the current gold standard of therapy is represented by the long-term use of hormonal treatments, especially low-dose oral contraceptives (COCs), progestins, gonadotropin-releasing hormone (GnRH) analogues and progestin-releasing intrauterine devices [66].

To avoid diagnostic delays, recent literature has focused on early diagnosis of endometriosis, by searching in severely symptomatic adolescent patients specific diagnostic signs of endometriosis [100,101]. In this subgroup of patients, hormonal therapy should be given at diagnosis. The purpose of this early treatment is to prevent chronic inflammation determined by repetitive ovulatory menstruation, thus worsening the disease, and, most importantly, exacerbating symptoms such as chronic pelvic pain. Indeed, chronic pelvic pain, when prolonged, can become centralized possibly giving rise to the onset of chronic overlapping pain conditions [102,103]. Recently, other studies focused on the assessment of symptomatic young patients without imaging signs of endometriosis. According to these authors, endometriosis should be suspected and promptly treated in symptomatic patients in the presence of some indicators, including early menarche and gastrointestinal symptoms [66,104,105].

The surgical treatment of endometriosis must be well adapted to the aspects of damage and the needs of the patient. The primary goal of modern laparoscopic surgery is the preservation of ovarian and reproductive function in young patients, and of only ovarian tissue in patients who do not desire further pregnancies. Laparoscopy is essential for a complete staging of the disease and allows for therapeutic actions. Medical treatment for endometriosis often involves hormonal therapies aimed to suppress estrogen production, thus limiting the growth of ectopic endometrial tissue. Combined estrogen–progestin therapies, such as oral contraceptives, inhibit ovulation and reduce circulating estrogen levels, thus limiting the stimulation of endometrial lesions. Progestins decrease the frequency and increase the amplitude of pulsatile gonadotropin-releasing hormone (GnRH) in the hypothalamus, thus decreasing the secretion of follicle stimulating hormone (FSH) and luteinizing hormone (LH). The long-term administration of progestins suppresses ovarian steroidogenesis, thus causing anovulation and reducing serum levels of ovarian hormones. The hypoestrogenism induced by these drugs causes decidualization of both eutopic and ectopic endometrium. Moreover, the association between changes in cytokine mRNA expression and nuclear receptors protein expression in response to progestins therapy may suggest a direct anti-inflammatory effect. GnRH antagonists directly inhibit gonadotropin release from the pituitary gland, thus creating a hypoestrogenic state able to shrink endometriotic lesions. Additionally, the levonorgestrel-releasing intrauterine device (IUD) provides a localized release of progestin, able to reduce the thickness of the endometrial lining and local inflammation associated with fewer systemic effects. Therefore, the IUD is a valuable option for long-term management. Each of these therapies has a unique mechanism of action and must be tailored according to the patients’ needs and tolerance. Currently, there are no guidelines specifying appropriate follow-up intervals to monitor the effectiveness of medical therapy in preventing disease progression or to check for new lesions in young women with typical symptoms. Despite these limitations, regular check-ups could be beneficial in evaluating the effectiveness of medical or surgical treatments, chosen according to the patient’s needs, in managing the disease and its associated symptoms.

### 3.3. IBD and Endometriosis: Shared Features

Endometriosis and IBD may share common symptoms, including abdominal pain, fatigue, infertility, menstrual irregularities and GI symptoms such as diarrhea and constipation [106]. In patients with deep endometriosis located in the rectum, chronic abdominal/pelvic pain may occur, thus mimicking symptoms related to UC or to CD involving the rectum and/or sigmoid colon. In subgroups of patients with endometriosis, particularly in patients with symptoms not clearly associated with the menstrual cycle (approximately 40%), the differential diagnosis between IBD and endometriosis may be clinically challenging.

The localization of intestinal endometriosis (IE) may influence the IE-related symptoms. In order to address this issue, Roman et al. in a three-arm cohort prospective study assessed the type and frequency of digestive symptoms in 116 patients with different localizations of pelvic endometriosis (superficial, deep or rectal DIE) [8]. Findings suggested that women with DIE infiltrating the rectum more likely experienced a significantly higher intensity and length of dysmenorrhea, while women with superficial endometriosis reported a more relevant deep dyspareunia (*p* < 0.01 for all). When compared with the other two groups, women with DIE of the rectum associated with stenosis were significantly more likely to experience constipation (*p* < 0.01), defecation pain (*p* = 0.04) and appetite disorders (*p* < 0.01). This subgroup of patients also showed an increased evacuation time (*p* < 0.01), an increased stool consistency without laxatives (*p* < 0.03) and a trend for a higher frequency of incomplete evacuation (*p* = 0.06) [8].

IE may mimic other inflammatory conditions such as IBD and diverticular disease both clinically and endoscopically. Even histologically, IE may determine mucosal architectural distortion and/or inflammation similar to IBD [107]. In a retrospective study, Guadagno et al. aimed to explore whether IE can mimic IBD and specific types of histological lesions shared by these two entities [9]. The study population included 100 consecutive, unselected cases of surgically resected IE [9]. A marked inflammatory and architectural mucosal changes similar to those observed in IBD was histologically detected only in a minority of patients. These findings suggested that IE may determine microscopic mucosal inflammation of the colon and IBD-like lesions as an epiphenomenon of endometriosis, while true IBD is observed only in a minority of cases [9].

Additionally, a recent study in women who never underwent surgery or hormonal therapy assessed the characteristics of symptoms of endometriosis [108]. Findings from this study suggested that specific localizations of endometriosis, including endometriosis of the USL, can cause bowel symptoms like constipation, diarrhoea and abdominal pain [108]. This is likely due to the close proximity of the USL to the rectum, causing inflammation that triggers bowel-related symptoms.

Very recently, one study suggested immunological and molecular characteristics shared by endometriosis and IBD [109]. Findings showed that both conditions are characterized by emphasized immune regulation and cell signaling, indicating the relevance of immune factors in the occurrence and progression of the two diseases [109]. However, this is preliminary evidence and the inflammatory response common to both IBD and endometriosis needs to be further investigated.

### 3.4. Impact of Endometriosis on Inflammatory Bowel Disease Risk

As for other diseases related to a dysregulation of the host immune response, a possible association between endometriosis and IBD has been investigated. Jess et al. in a retrospective study examined all women with a diagnosis of endometriosis followed by a diagnosis of IBD over a 30-year period (1977–2007) [7]. Patients were recruited from the Danish National Hospital Register and those with a previous diagnosis of IBD were excluded. Analyses were restricted to the period from 1980 to 2007 in women who underwent surgery related to endometriosis. The cohort included 37,661 women with endometriosis (mean age of 38.6 years), 87% of whom were treated as inpatients. Overall, findings indicate an association between IBD and endometriosis. A diagnosis of UC was made in 228 patients (SIR 1.5, [1.3–1.7]) and of CD in 92 patients (SIR 1.6 [1.3–2.0]), with a combined risk of IBD of 1.5 [1.4–1.7]. The association between IBD and endometriosis was stronger in women with surgically verified endometriosis (SIR 1.8 in UC and 1.7 in CD). The risk of UC differed in relation to the age at diagnosis of endometriosis, as the highest risk was observed in women with a diagnosis of endometriosis at the age between 25–34 years (SIR 2.0 [1.6 to 2.4]). The risk of CD was higher in patients with endometriosis diagnosed before the age of 25 years (SIR 2.0 [1.2 to 3.5]), although this variable was not significantly associated with the risk of concomitant CD. The mean time interval from the diagnosis of endometriosis to the diagnosis of IBD did not significantly differ between UC or CD. Compared with the general Danish population, a 50% increased risk of IBD was observed in women with endometriosis [7].

In a retrospective cross-sectional study including 781,571 adult women of which 6647 were diagnosed with endometriosis, 25,425 with fibromyalgia and 401 with both conditions, the association between endometriosis, fibromyalgia and IBD was assessed [86]. Women with concomitant endometriosis and fibromyalgia showed a significantly higher prevalence of IBD in women with vs. without concomitant endometriosis/fibromyalgia (6.2% vs. 1%; *p* < 0.001) [13].

Differently, in a case-control study including 148 women with endometriosis and 150 controls without endometriosis, IBD was diagnosed in 5 patients with endometriosis and in none of the other group (*p* = 0.07) [12].

A Danish retrospective study assessed the risk of ectopic pregnancy in pregnancies of women with IBD compared with those without IBD over a 22-year period [11]. The authors investigated the risk of an ectopic pregnancy in women with or without IBD. This study included a large cohort of patients (7548 pregnancies in the UC cohort, 6731 pregnancies in the CD cohort and 1,832,732 pregnancies in the non-IBD cohort). Among findings of the study, the authors reported that endometriosis was more prevalent in pregnant women with vs. without IBD [11].

Finally, in a recent nested case-control study, endometriosis was detected in 25 out of 35 IBD patients with compatible symptoms (71%) [14].

### 3.5. Characteristics of IBD in Patients with Concomitant Endometriosis

Whether patients with endometriosis show particular characteristics of IBD has been more recently investigated. However, limited data are currently available.

In 2000, a Belgian case-report reported an intra-operatory diagnosis of intestinal endometriosis of the ileum (n = 6), colon (n = 1) or ileum and rectum (n = 1), in eight female patients who underwent surgery for complicated CD [3]. Concomitant lesions related to CD were also confirmed in endometriosis-free intestinal areas [3].

In 2016, a larger American case-control study, including 51 cases of patients with concomitant IBD and endometriosis, aimed to assess whether women with concurrent endometriosis and IBD have a unique phenotype and worse clinical outcomes than IBD patients without endometriosis [10]. Cases and controls were matched for age and IBD type. Among CD patients with concomitant surgically confirmed endometriosis there was a higher risk of stricturing disease when compared with CD controls (OR 11.8 [2.03–69.0]). Differently, there was no difference in terms of phenotype in UC patients with concomitant surgically confirmed endometriosis. Regarding the clinical outcome, there was no significant difference in terms of IBD-related medical or surgical treatments when stratified according to IBD type [10]. Therefore, currently available evidence does not support an association between endometriosis and IBD characteristics.

### 3.6. Characteristics of Endometriosis in Patients with Concomitant IBD

Whether patients with IBD and concomitant endometriosis show specific localizations of endometriosis foci has been investigated in few studies. In a nested case-control study, the possible association between IBD and endometriosis, and also possible differences in terms of characteristics, type and sites of endometriotic lesions in matched patients with or without IBD, was evaluated [14]. In a study population including 125 patients with endometriosis, the age at diagnosis of endometriosis was higher in IBD patients with vs. without concomitant IBD (38 [25–53] vs. 30 [22–52]; *p* = 0.01). Dyschezia and dyspareunia were more frequently observed in cases than in controls (11 [44%] vs. 17 [17%]; *p* = 0.008 and 19 [76%] vs. 51 [51%]; *p* = 0.04). DIE was detected by TVS in all cases (105 [100%]), and its frequency was higher in cases than in controls (25 [100%] vs. 80 [80%]; *p* = 0.03) as also posterior adenomyosis (19 [76%] vs. 48 [48%]; *p* = 0.02). Therefore, this recent preliminary evidence suggests that IBD may be associated with DIE and characterized by pelvic symptoms such as dyschezia and dyspareunia [14].

### 3.7. Treatments for Endometriosis in Patients with IBD

An increased risk of developing IBD has been suggested but not confirmed in patients with endometriosis treated with hormonal therapy, although data in this respect are not conclusive [110,111,112]. The mechanism through which contraceptive formulations, including dose and treatment duration, may potentially affect the risk of IBD is unknown. The potential role of estrogens as modulators of inflammation and immunity interfering with the colonic mucosa barrier has been proposed. Thrombotic risk associated with the use of OCPs has also been hypothesized to be involved in tissue damage in IBD, particularly in CD [113,114,115,116].

In women with a preexisting higher thrombotic risk, the use of these drugs should be tailored on a patients’ basis [117]. This is also in relation to the higher risk of thrombotic events in active IBD, including occlusive disease in the vessels supplying the involved CD areas of subgroups of patients. Although conflicting, current evidence also does not suggest that OCPs significantly increase the risk of IBD flares, as reported in a systematic review [118].

Differences in terms of type, dose and duration of oral contraceptive pill (OCP), IBD characteristics, type (UC or CD), severity and extent may well account for discrepancies regarding the potential role of these drugs as a risk factor for IBD. Moreover, by our knowledge, no data from controlled trials are currently available. Findings regarding the potential dose–response relationship between OCP exposure and IBD development are indeed conflicting. [111]. Boyko et al. reported an increased UC risk using higher potency estrogen compounds (OCPs containing ≥ 32 µg of estrogen) [119]. In contrast, Vahedi et al. reported no correlation between low- vs. high-dose OCPs and the development of UC [120,121,122,123]. Pasvol et al. suggested an increased IBD risk after long-term treatment with combined OCPs [124]. Progestogen-only pills had a modest association with UC (OR 1.35 [1.12–1.64]), and no effect on CD risk (OR 1.09 [0.84–1.40]). There was no association between parenteral progestogen-only contraception and IBD [124]. A different study focused on the potential effects of treatments for endometriosis on an IBD course, and vice versa. In this study, a subgroup of 20 out of the 25 (80%) IBD patients with concomitant endometriosis was treated with hormonal therapy, which induced amenorrhea [14]. Treatments included progestin and estro-progestins given continuously and for a long period since endometriosis is a chronic disease like IBD. In the case of adenomyosis, progestin-releasing intrauterine devices were indicated. GnRh analogues and antagonist, due to their potential side effects, were given only when hormonal treatment failed. At 6 months, all the 12 IBD patients followed-up for 6 months after hormonal therapy showed clinical improvement in terms of both abdominal pain and endometriosis-related symptoms [14]. However, findings from this study are limited by the short follow-up and the small study population. Overall, further research is therefore required in order to clarify the possible relationship between IBD risk and OCPs, which is however currently not supported by available evidence.

To date, no treatments are currently effective for simultaneously treating IBD and endometriosis. In patients with both conditions, current evidence does not support the need of prioritizing one over the other disease. Each of the two diseases should be treated according to the most updated and dedicated guidelines, as the rate of AEs appear not to be increased when using treatments for both conditions. In rare cases, the choice to prioritize one of the two diseases should rely on a patient-to-patient basis, depending on the occurrence of special urgent situations such as ASUC and complicated CD or severe DIE. The need of surgery for endometriosis may, however, require a temporary discontinuation of immunomodulatory treatments in the perioperative period, as before other surgical indications. In this respect, surgical procedures for endometriosis may determine technical difficulties for the surgeon in the case of patients with previous major surgery for IBD (i.e., proctocolectomy with ileal pouch or ileorectal anastomosis, previous pelvic abscesses, etc.).

### 3.8. Biologic Therapies for Treating Endometriosis

Currently available medical treatments for endometriosis are mainly focused on estrogen synthesis suppression, induction of atrophy of ectopic (displaced or mispositioned) endometrial implants or stopping the cycle and menstrual bleeding. Oral contraceptives, androgenic agents, progestins and GnRH analogues have all been used successfully for treating endometriosis [66].

Since the host immune response is reputed to be involved in the pathogenesis of endometriosis, immunomodulatory treatments have been tested in order to reduce the pain and infertility without inhibiting the ovulation [125].

Infliximab is a chimeric monoclonal antibody that binds to both soluble and membrane-bound TNF-α, approved by the Food and Drug Administration (FDA) for treating IBD patients [45,50,126]. This is the only biologic approved for IBD with available data when used for endometriosis. In a randomized placebo-controlled trial, patients with deep endometriosis were treated with either infliximab at the same dose used for IBD (5 mg/kg) or placebo [125]. Excisional surgery was performed after 3 months and patients were followed-up for an additional 6 months. The primary end-point was pain assessed at each visit by the clinician and on a daily basis by the patient. Findings showed that pain severity decreased during treatment by 30% in both the placebo (*p* < 0.001) and the infliximab group (*p* < 0.001). After surgery, pain scores decreased in both groups to ≤20% of the initial value. According to these preliminary findings, the efficacy of infliximab in patients with endometriosis appears not relevant [125].

## 4. Discussion

Differentiating IBD from endometriosis in patients with compatible demographic characteristics requires an accurate clinical history and assessment, followed by diagnostic procedures. The more frequent symptoms associated with uncomplicated IBD include chronic recurrent abdominal pain (mainly in CD), diarrhea with or without macroscopic blood, frequent weight loss, fatigue, fever and extraintestinal manifestations. In patients with endometriosis, chronic pelvic pain, dysmenorrhea, dyspareunia, dyschezia, dysuria and infertility represent the more typical symptoms. Blood chemistry may detect iron-deficiency anemia (IDA) in both conditions, while macrocytic anemia due to B12 deficiency may be observed only in IBD (CD), but not in endometriosis patients. Patients with IBD, but not with endometriosis, may also show several markers of inflammation (i.e., elevated ESR, CRP, fecal calprotectin), signs or symptoms related to malabsorption or related to inflammatory-related hypercatabolism (hypocholesterolemia, hypoalbuminemia, folate deficiency, leucopenia), together with electrolyte imbalance (hypokalemia, hyponatriemia, hypocloremia), up to sarcopenia. In patients with suspected IBD on the basis of the abovementioned criteria, diagnostic procedures include ileocolonscopy with biopsies and small bowel imaging (ultrasonography, entero-CT or entero-MRI). In subgroups of patients, an abdominal CT scan and pelvic MRI may also be required. In case of suspected endometriosis according to clinical assessment, gynecological evaluation, TVU and/or pelvic MRI in experienced hands are indicated.

Regarding challenges in the differential diagnosis between IBD and endometriosis, both gastroenterologists and gynecologists should be aware of the “red flags” suggesting the need to consider an underlying diagnosis of these conditions. The knowledge of these red flags allows a proper and timely referral to IBD- or endometriosis-dedicated specialists. This in order to overcome diagnostic difficulties also including a possible differential or concomitant diagnosis. During a gastroenterological outpatient visit, the search for the abovementioned typical symptoms observed in the case of endometriosis should suggest the need for a gynecological evaluation. In a case-control study from our group, a diagnosis of concomitant endometriosis was indeed made in 71% of IBD patients with compatible symptoms [14]. Differently, chronic pelvic pain related to the menstrual cycle should raise the suspicion of a diagnosis of endometriosis rather than IBD. Vice versa, a consensus paper proposed red flags, suggesting a potential diagnosis of IBD requiring referral to a gastroenterologist in patients with spondyloarthritis. These include the detection of one major criterium among chronic diarrhea, rectal bleeding, perianal fistula/abscess, chronic abdominal pain and nocturnal symptoms or three minor criteria among oral aphtosis, fever, anemia, family history of IBD and weight loss [127]. Taking into account the potential difficulties related to a proper and timely diagnosis of IBD and/or endometriosis, a multidisciplinary approach including dedicated clinicians is required.

IBD and endometriosis may not only share signs and symptoms such as abdominal pain, diarrhea, hematochezia, tenesmus and IDA, but also a relapsing course and the possible involvement of the same target organs. Recent preliminary observations suggest that endometriosis and IBD may even share some molecular mechanisms [109]. Some of the pathogenetic mechanisms involving the host immune regulation and immune cell signaling have indeed been hypothesized to be common in these two conditions. This evidence, showing a dysregulation of the immune response in both IBD and endometriosis, suggests the potential efficacy of immunomodulators in these diseases. Different to endometriosis, the inflammatory pathways leading to inflammation in IBD have been more clearly described, even though not completely defined [128,129]. In IBD, since the 1970s, immunomodulators (thiopurines) have been shown to be one of the most effective treatments for steroid-dependent or chronically active IBD patients [15,16,32,45]. Since the late 1990s, biologic treatments, initially including only TNF-α antagonists, have been developed [28,42,50]. These treatments currently show a high efficacy, allowing not only the achievement of clinical remission, but also the improvement or healing of the lesions in subgroups of patients [28,42,50]. Among the several immunomodulatory drugs approved for IBD, only the TNF-α antagonist infliximab has also been used for treating endometriosis, with disappointing results [125]. Moreover, other treatments such as pentoxifylline gave conflictual results in terms of efficacy in patients with endometriosis [4]. Therefore, despite the abovementioned preliminary observations regarding the pathogenesis of endometriosis, current evidence does not support the usefulness of immunomodulators common to IBD for treating endometriosis.

**Figure 4:**
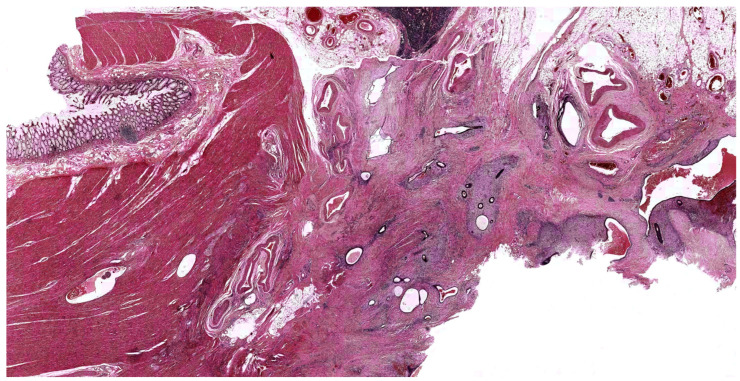
Microscopic aspect of the bowel wall from one patient with ulcerative colitis, showing foci of endometriosis within the muscular layer and the perivisceral fat tissue. Hematoxylin and eosin stain (magnification 10×).

A concomitant diagnosis of IBD and endometriosis has been described in several observations [3,130,131] (Figure 4). This is expected as both diseases, particularly endometriosis, are not rare [1,15]. More interestingly, however, is that current preliminary evidence suggests a higher risk of IBD in patients with endometriosis [7]. The large nationwide Danish cohort study indeed reported a 50% increased risk of IBD in women with vs. without endometriosis [7]. The observation of a persistently increased risk of endometriosis (non IBD) more than 20 years after a diagnosis of IBD also strengthens the possibility of a true association rather than reflecting a diagnostic delay. Whether the increased risk of IBD may be related to treatments for endometriosis, including oral contraceptives, gestagens, progestins, GnRh agonists and, to some extent, NSAIDs and anti-COX2, can be only hypothesized [9]. Oral contraceptive use has been associated with the development of IBD, even though the reason for this observation is still undefined.

To date, no treatments are available that can simultaneously address IBD and endometriosis. In patients with both diseases, current evidence does not support the need of prioritizing one over the other. Indeed, each disease should be treated according to the most updated guidelines available with specific drugs, which often do not interfere with each other. When required, the choice to prioritize one disease over the other should rely on a patient-to-patient basis, depending on the occurrence of special urgent situations such as ASUC and complicated CD or severe DIE. Treating endometriosis currently presents significant challenges, in terms of both medical and nonmedical approaches. Medical treatments aim to suppress endometrial tissue growth and to reduce related symptoms. However, these treatments often determine side effects including bone loss and mood disturbances, thus reducing compliance in the long-term. Furthermore, current medical treatments are currently not curative, as symptoms recurrence is common after treatment discontinuation. Surgical approaches, although effective for symptom relief, carry risks of complications, recurrence and impaired fertility. Noninvasive options, including lifestyle modifications and alternative therapies (i.e., acupuncture, dietary changes, etc.), have a lack of scientific evidence supporting their efficacy. Thus, the current management of endometriosis requires a comprehensive, multidisciplinary approach that balances symptom relief, quality of life and long-term management, while addressing the need for personalized treatments and improved patient care. This issue must be urgently addressed in patients with both endometriosis and IBD, as they often require multiple therapies to manage their symptoms. This may improve their quality of life and acceptance of both diseases. However, it may increase the risk of experiencing additional side effects, possibly leading to reduced compliance and even to treatment discontinuation.

The diagnosis of endometriosis still often relies on surgical findings. During the last few years, noninvasive diagnostic methods such as MRI and TVS in experienced hands can be considered as a valid alternative to surgery [84]. Since in patients with endometriosis, IBD-like lesions may be detected [9], the concomitant diagnosis of IBD should be adequately reassessed in subgroups of patients.

Symptoms related to endometriosis and IBD may differ according to the phenotype and localization of the diseases. In a case-control study from our group [14], dyschezia and dyspareunia in patients with endometriosis were significantly more frequent in patients with IBD than in the controls. A higher frequency of DIE was reported in patients with IBD than in the non-IBD controls, suggesting that symptoms such as tenesmus and chronic pelvic pain in IBD may be related to endometriotic nodules localized in the sigmoid colon and rectum. In the presence of these symptoms, particularly in IBD patients refractory to conventional treatments, a concomitant diagnosis of DIE located in the distal colon should be carefully searched in order to exclude a concomitant diagnosis of endometriosis. The observed higher frequency of posterior endometriosis in the tested population (*p* = 0.04) further supports that diagnosing endometriosis in IBD patients requires not only the search for specific symptoms, but also an assessment by dedicated gynecologists [14].

## 5. Conclusions

When considering current evidence regarding the possible association between endometriosis and IBD, limitations include the small number of studies investigating this issue, the small study populations investigated and the study design. Further studies are therefore needed in order to evaluate the potential association and relationships between IBD and endometriosis and also to identify red flags for concomitant diseases. Characteristics of both IBD and endometriosis in patients with concomitant conditions also needs to be further evaluated. Whether new treatments may improve the outcome of both conditions represents an additional issue. Current preliminary findings, however, suggest the need of a careful search for concomitant endometriosis in subgroups of patients with IBD showing compatible symptoms. Accordingly, gynecologists should be aware of a potential risk of IBD in patients with GI symptoms, as tenesmus and rectal bleeding are not responsive to hormonal treatments for endometriosis. The identification of defined red-flags for IBD- and endometriosis-dedicated clinicians, as proposed for other chronic diseases, may help to discriminate symptoms related to each condition and to achieve a proper diagnosis.

## 6. Future Perspectives

The reported few observations suggest that a multidisciplinary approach including dedicated gastroenterologists and gynecologists is required for a proper search for IBD and endometriosis in subgroups of patients. This approach may avoid diagnostic delays and potential ineffective overtreatments for these conditions, in order to optimize the outcome and the quality of life of the affected patients.

## Figures and Tables

**Table 1:** Studies involving patients with endometriosis and/or inflammatory bowel diseases fulfilling the inclusion criteria: overview.

Author	Year	Study Design	Patients with Endometriosis Only (n=)	Patients with IBD Only (n=)	Patients with Both IBD and Endometriosis (n=)	Surgical Diagnosis of Endometriosis Only	Primary Aim	Primary Outcome
Craninx et al. [3]	2000	Case Series	0	319 (CD)	8	Yes	Endometriosis in CD patients undergoing surgery	Endometriosis diagnosed in the resected bowel of patients with CD
Jess et al. [7]	2012	Cohort study	37,341	0	320 (92 CD, 228 UC)	Yes	To assess the risk of CD and UC in a nationwide cohort of endometriosis patients	Increased risk of IBD, CD and UC in patients with vs. without endometriosis (SIR 1.5 [1.4–1.7], SIR 1.5 [1.3–1.7], SIR 1.6 [1.3–2.0])
Roman et al. [8]	2012	3-arm cohort prospective study	116	0	0	Yes	Digestive symptoms in patients with different endometriosis sites	Low frequency of rectal stenosis (26.4%) in rectal endometriosis
Guadagno et al. [9]	2015	Retrospective observational study	100	0	0	No	Frequency of IBD-like histology in intestinal endometriosis	IBD-histological mucosal features in 3% of samples
Lee et al. [10]	2016	Case-control study	0	102 (54 CD 48 UC)	51 (28 CD, 23 UC)	No	Phenotype and prognosis of IBD in endometriosis patients	Comparable race, age at IBD diagnosis, IBD or UC duration, UC phenotype
de Silva et al. [11]	2018	Case-control study	n.a.	14,000 (7401 UC, 6584 CD)	297 pregnancies with endometriosis (150 UC, 147 CD)	No	Risk of ectopic pregnancy in patients with vs. without IBD	Higher risk of ectopic pregnancy, in CD (but not in UC) vs. no IBD (CD: OR 1.23; [1.01–1.49]; UC: OR 0.98 [0.80–1.20])
Porpora et al. [12]	2019	Case-control study	143	0	5	No	Prevalence of IMIDs in patients with/without endometriosis	Higher prevalence of IBD in patients with/without endometriosis (5/148 vs. 0/150; *p* = 0.07)
Greenbaum et al. [13]	2019	Retrospective cross-sectional study	6883	0	165	No	Prevalence and association between endometriosis, fibromyalgia and IMIDs	Higher IBD prevalence in patients with/without endometriosis and fibromyalgia (6.2% vs. 1%; *p* < 0.001)
Neri et al. [14]	2023	Case control study	100	0	25 (13 UC, 12 CD)	No	Symptoms, type and site of endometriosis in patients with/without IBD	Dyspareunia, dyschezia, DIE and posterior adenomyosis more frequent in IBD vs. controls (n [%]: 25 [73.7%] vs. 26 [45.6%]; *p* = 0.03; 25 [100%] vs. 80 [80%]; *p* = 0.03 and 19 [76%] vs. 48 [48%] *p* = 0.02)

Abbreviations: n = number, IBD = inflammatory bowel disease; CD = Crohn’s disease; UC = ulcerative colitis; n.a. = not applicable; DIE = deep infiltrating endometriosis; IMIDs: immuno-mediated diseases; SIR = standardized incidence ratio; OR = odds ratio.
